# Malaria, Urogenital Schistosomiasis, and Anaemia in Pregnant Ghanaian Women

**DOI:** 10.1155/2023/7500676

**Published:** 2023-09-29

**Authors:** Naa Adjeley Frempong, Charity Ahiabor, William K. Anyan, Atikatou Mama, Kwadwo Asamoah Kusi, Michael F. Ofori, Bright Adu, Alex Yaw Debrah, Abraham K. Anang, Nicaise T. Ndam, David Courtin

**Affiliations:** ^1^Clinical Microbiology Department, Kwame Nkrumah University of Science and Technology, Ghana; ^2^Parasitology Department, Noguchi Memorial Institute for Medical Research, College of Health Sciences, University of Ghana, Legon, Ghana; ^3^Science Laboratory Technology, Accra Technical University, Ghana; ^4^Inserm U 1016, Institut Cochin, Université de Paris, 75014, France; ^5^Immunology Department, Noguchi Memorial Institute for Medical Research, College of Health Sciences, University of Ghana, Legon, Ghana; ^6^Faculty of Health Sciences, Kwame Nkrumah University of Science and Technology, Ghana; ^7^Institute of Environment and Sanitation Studies(IESS), University of Ghana, Legon, Ghana; ^8^Université Paris Cité, MERIT, IRD, Paris, France

## Abstract

**Background:**

Anaemia is common in sub-Saharan Africa, and parasitic infections could worsen its burden during pregnancy. Moreover, women become susceptible to malaria during pregnancy. We investigated *Plasmodium falciparum* (*P. falciparum*) and *Schistosoma haematobium* (*S. haematobium*) infections and determined their association with anaemia during pregnancy.

**Methods:**

A cross-sectional study involving 707 pregnant women attending antenatal care visits (ANC) and 446 at delivery was conducted in Battor and Adidome hospitals. Pregnant women were screened by microscopy and qPCR for *P. falciparum* and *S. haematobium* infections. Haemoglobin (Hb) levels were determined, and most participants received intermittent preventive treatment during pregnancy (IPTp) during ANC till delivery. Regression analyses were performed for associations between parasite infection and anaemia.

**Results:**

*P. falciparum* microscopy prevalence at ANC and delivery was 8% and 2%, respectively, and by PCR 24% at ANC and 12% at delivery. Anaemia prevalence at ANC was 52% and 49% at delivery. There was an increased risk of anaemia with *P. falciparum* infection (aOR = 1.92; *p* = 0.04). IPTp (*p* = 0.003) and age (*p* = 0.004) were associated with increased Hb levels at delivery. *S. haematobium* prevalence by microscopy was 4% at ANC and 2% at delivery. No significant correlation between *S. haematobium* and Hb levels was observed (coef. = −0.62 g/dl; *p* = 0.07).

**Conclusion:**

High anaemia prevalence was observed during pregnancy, and *P. falciparum* infection was associated with anaemia at ANC. Low *S. haematobium* prevalence could be attributed to previous praziquantel treatment during mass drug administration. Routine diagnosis and treatment of *S. haematobium* infections in endemic areas could be initiated to reduce schistosomiasis during pregnancy.

## 1. Background

About 1.5 billion infections resulting from malaria and helminthiases contribute to pregnancy-related morbidity and mortality [1], exposing the foetus to several complications including anaemia, still birth, intrauterine growth retardation, and low birth weight infants [2, 3]. Malaria and schistosomiasis are widespread parasitic infections in tropical and subtropical regions including Ghana [4, 5] and pose a double disease burden during pregnancy in coendemic areas. Disease burden seems to be greater in women of reproductive age [6], often leading to lowered immunity, increased susceptibility to other infections, and poor pregnancy outcomes [7, 8]. Another critical complication of malaria and schistosomiasis is anaemia, which could have severe consequences on the health of the mother [9].

The World Health Organization (WHO) estimates that out of 40 million pregnancies reported in sub-Saharan Africa, 13.3 million cases have been linked to pregnancy-associated malaria (PAM) [10]. Despite enormous control and prevention strategies for the fight against malaria, it remains a disease of public health importance in Ghana with a total of 5 million cases including at least 390,000 hospital admissions [11]. *Plasmodium falciparum* infections contribute to 18% of outpatient department cases, leading to 14% of hospital admissions and 3% maternal deaths [12]. As part of the efforts aimed at fighting malaria, the program hopes to reduce malaria mortality by 90% and malaria incidence by 50% by 2025 [11]. WHO estimates that 40% of pregnant women worldwide are anaemic [13, 14] and the situation is no different in Ghana as it stands at 47% ranging from 37 to 53% [15]. Malaria has been identified as an important independent risk factor for anaemia [16] and contributes to about 3-15% of anaemia cases and 25% of the total severe anaemia in pregnant women from malaria endemic countries [16]. The destruction of *P. falciparum*-infected erythrocytes [17] and defective erythropoiesis in the bone marrow could lead to anaemia [16].

Schistosomiasis, another known cause of anaemia [18], affects nearly 200 million people worldwide with considerable economic impact and morbidity [6, 7, 19]. As a disease of poverty, 97% of schistosomiasis infections and 85% of at-risk population live in Africa [20]. *Schistosoma haematobium* prevalence in pregnancy ranges from 0.30 to 4.53% in Northern Ghana and Dangme East District [21, 22]. A recent systematic review and meta-analysis study linked schistosomiasis to anaemia, while mass drug administration (MDA) among pregnant women reduced anaemia by 23% [18, 23]. In *S. haematobium* infections, the human host loses blood and iron when *S. haematobium* eggs with terminal spines pass through the urogenital tract. Additionally, inflammation in response to dislodged eggs in host tissues could be the common cause of low haemoglobin (Hb) levels and anaemia [24].

A problem of underreported data on schistosomiasis exists in pregnancy. Therefore, it is necessary to generate accurate data on pregnant women who form a high-risk group to improve and guide schistosomiasis control strategies. Furthermore, a meta-analysis exposed a primary concern of scanty data on schistosomiasis outcomes in pregnancy [18]. WHO highly recommends anthelmintic therapy after the first trimester of pregnancy [25–27] to improve maternal and infant health [7, 14]. In Ghana, although on-going programs exist to control schistosomiasis and targeting elimination in the future [28], the neglected tropical disease program reports suboptimal treatment during pregnancy.

Malaria and schistosomiasis are coendemic in Ghana [4], and enormous malaria control efforts have been invested during pregnancy, including intermittent preventive treatment during pregnancy (IPTp) and insecticide treated nets (ITNs) during first antenatal care visit (ANC) [29–31]. Furthermore, intensive case search by screening and treatment exists for malaria prevention during pregnancy at ANC [32]. However, routine schistosomiasis diagnosis and preventive chemotherapy are not fully exhaustive. We investigated prevalence of malaria and urogenital schistosomiasis and associated anaemia in two study hospitals at ANC and at the point of delivery.

## 2. Methods

### 2.1. Ethics

Ethical clearance was obtained from the Ghana Health Service (GHS) with protocol number GHS-ERC 06/06/16 and Noguchi Memorial Institute for Medical Research (NMIMR-IRB CPN 071/15-16).

### 2.2. Study Area and Population

This study was carried out in Adidome Government Hospital (AH) and Battor Catholic Hospital (BH), located in Adidome and Battor which are capital towns of Central and North Tongu Districts of the Volta Region, respectively, in Ghana [33]. The Tongu District is located in southeastern Ghana and mainly inhabited by the Ewe tribe, with farming and fishing as major occupations. A total of 110,891 people inhabit North Tongu and 83,803 Central Tongu District [34]. The presence of the Volta River and its numerous tributaries, dams, and dugouts in the district constitute a great potential for irrigation farming and fishing. Heavy reliance of these dugouts for fresh water source and income generation activities makes it a prime environment for schistosomiasis transmission through human contact with infected vector snails [35]. In the Volta Region, schistosomiasis is endemic [36], with a prevalence of 10% in children [37], and malaria prevalence has been ranked the third highest in 16 administrative regions [38].

### 2.3. Study Design

A cross-sectional study was conducted in women aged 16–45 years, attending ANC for the first time and the maternity ward for delivery in AH and BH from November 2016 to March 2019. Study participants had positive urine pregnancy test, ultrasound scan of foetus, and were HIV negative. Women with history of a debilitating condition in pregnancy and those receiving anthelmintics and malaria treatment prior to enrolment at ANC were exempted from our study. After consent was given by participants, questionnaire data was captured using CSPro (V6.2)-based electronic tablets while other relevant disease prevention tools that guided the study were gathered from hospital records. Each participant provided urine and blood samples for *S. haematobium* and *P. falciparum* diagnosis, respectively. During the first ANC, biological samples were collected before the administration of IPTp. At the point of delivery, the number of IPTp doses was gathered from hospital records.

### 2.4. Sample Collection and Analyses

#### 2.4.1. Blood

Hb levels of pregnant women were determined using a Sysmex haematology analyser [39] at the time of sampling at ANC and delivery. A thick blood film was prepared for each sample, stained with Giemsa, and observed under the microscope to determine *Plasmodium* spp. [40, 41]. *Plasmodium falciparum* parasites were counted per 200 or 500 white blood cells (WBC) in thick blood film, and parasite density was estimated per microlitre (*μ*l) of blood. A minimum of 100 fields was counted before each slide was determined as negative [42, 43].

Blood was blotted onto filter paper, and DNA was extracted and amplified using quantitative PCR (qPCR) for *P. falciparum* determination according to previous methods [44–46].

#### 2.4.2. Urine

Each of 40 ml urine collected was gently swirled to ensure uniform distribution of parasite ova, and 10 ml of urine was drawn and passed through a holder fitted with filter membrane [47]. Subsequently, the filter membranes were placed on a microscope slide to observe and count *S. haematobium* eggs under a microscope at a magnification of 10x [48, 49]. For quality control, 10 out of 100 prepared slides were randomly selected from each box by another independent trained microscopist for examination [50]. *S. haematobium* infection intensity was expressed as the number of eggs per 10 ml urine with a threshold (<49 eggs/10 ml of urine) for light intensity and (≥50/10 ml) for heavy intensity [48]. Anaemia in pregnancy was classified using WHO guidelines into normal (>11), mild (10-10.99), moderate (7.0-9.99), and severe (<7) groups [51].

### 2.5. Statistical Analysis

Statistical analyses were performed using SPSS version 22 (IBM). A *Plasmodium* parasite classification was applied by estimating the median parasitaemia of pregnant women at a threshold of 2,500 *μ*l parasites/blood. Bivariable analyses were performed using the nonparametric chi-square or Kruskal-Wallis test for associations between variables and Hb. Multivariable linear regression analyses were performed for variables that showed a *p* value < 0.2 in a bivariable analyses. A logistic regression model determined factors including parasitic infections associated with anaemia in pregnancy.

## 3. Results

### 3.1. Characteristics of Participants

The characteristics of study participants at ANC and delivery have been summarized (Table 1). A total of 707 pregnant women were recruited at ANC (AH = 393, BH = 314) and 446 at delivery (AH = 330, BH = 116). Pregnant women in BH were older with significantly higher Hb levels compared to AH. These women from BH attended their first ANC much later in their pregnancy than those in AH. At delivery, many pregnant women (95% from AH and 100% from BH) reported to have been treated with IPTp during pregnancy. Similarly, a high proportion of them (90% and 96%) indicated that they slept in bed nets at ANC and delivery. Malaria prevalence in AH was significantly higher than BH at ANC and delivery, but there was no statistical difference in urogenital schistosomiasis prevalence in the two hospitals at ANC.

### 3.2. Malaria and Urogenital Schistosomiasis Prevalence among Pregnant Women

Malaria prevalence by microscopy was 8% (54/677) at ANC and 2% (9/379) at delivery and by PCR was 24% (160/677) at ANC and 12% (44/379) at delivery. Median parasitaemia was 2,020  parasites/*μ*l of blood. At ANC, the geometric mean parasitaemia for *P. falciparum*-infected women was 1,995 (95% CI 1,259-3,162) parasites/*μ*l. Urogenital schistosomiasis prevalence at ANC at AH was 4% (14/348) and at BH 3% (11/324); 0.7% (5/662) had *P. falciparum* and *S. haematobium* coinfections. From a total of 192 urine samples collected at delivery, only 2% were positive for *S. haematobium* in BH (3/192).

Out of 28 pregnant women (25 at ANC and 3 at delivery) who had *S. haematobium* infections, the mean egg intensity was 13 eggs/10 ml (1-61 eggs/ml). Only one participant was classified as high intensity (61 eggs/10 ml) by standard protocols [25] with the remaining 27 falling within low intensity (<50 eggs/10 ml) category.

### 3.3. Anaemia and Parasitic Infections in Pregnant Women

Anaemia status of pregnant women has been summarized (Table 2). Anaemia prevalence at ANC for women at AH and BH was 55% (217/392) and 47% (147/314), respectively. At delivery, anaemia prevalence was 50% at AH and 48% at BH. All severe cases of anaemia were observed in AH, and no pregnant woman reported with severe anaemia at BH, either at ANC or delivery.

*P. falciparum* infection was significantly related with decreased Hb levels (*p* = 0.002), and age was associated with an increased level of Hb (*p* < 0.001, linear regression model) in pregnant women from ANC (Table 3). At delivery, Hb levels for women who had taken IPTp during their pregnancy were significantly higher than those who had not been treated (*p* = 0.003). Age was also associated with an increased Hb level at delivery (*p* = 0.004). It was also observed that more than two (≥2) IPTp doses received during pregnancy were positively associated with increased Hb levels compared to only one dose.

Subsequently, Hb levels of pregnant women were classified using WHO recommendations for anaemia. Women infected with *P. falciparum* had a higher risk of being anaemic at ANC (aOR = 1.92, *p* = 0.04). The odds of pregnant women developing anaemia as they get older was lowered (aOR = 0.95, *p* = 0.001) (Table 4). Furthermore, we grouped anaemia status of pregnant women into mild, moderate, and severe, and *P. falciparum* infection (aOR: 15.19, *p* = 0.006) and age (aOR: 0.79, *p* = 0.037) were associated with severe anaemia when compared to women without anaemia (reference group). Similarly, malaria (aOR: 2.26, *p* = 0.07) and age (aOR: 0.94, *p* < 0.0001) were associated with the risk of becoming moderately anaemic at ANC. At delivery, IPTp use (aOR: 0.79, *p* = 0.03) was significantly associated with protection against mild anaemia. No association was observed between *P. falciparum* parasitaemia levels and anaemia (*p* value, 0.59).

*S. haematobium*-infected women at ANC presented lower Hb levels than uninfected women (coef.: -0.62 g/dl; 95% CI (-1.26; 0.06)), although this was not statistically significant (*p* = 0.07). In a multivariable linear model, this effect remained not significant (*p* = 0.21) when adjusted for other covariates (Table 3). Nonetheless, the only participant with high *S. haematobium* infection intensity had the least Hb level (5.4 g/dl) within severe anaemia range (<7.0 g/dl).

## 4. Discussion

Anaemia is a major health concern during pregnancy [22, 52], and the presence of malaria and schistosomiasis could increase its burden [18, 52]. Innovative strategies targeted at pregnancy-associated malaria (PAM) include prompt diagnosis and preventive treatment at ANC [31, 53] to achieve a “zero malaria” target by 2030 [54, 55]. Schistosomiasis is one of the neglected tropical diseases (NTDs) targeted for elimination as a public health problem by 2030 [19, 25]. However, in Ghana, pregnant women are exempted from MDA, and there seems to be no routine diagnosis and treatment during ANC programs. We investigated malaria- and schistosomiasis-related anaemia in women attending ANC and delivery in AH and BH.

The Ghana Demographic Health Survey Report reported that 45% of pregnant women were anaemic in Ghana [56]. Our study recorded that over 50% of pregnant women were anaemic which is a clear indication of a higher prevalence of anaemia in pregnant women in our study areas than the national average. Anaemia prevalence studies conducted in Tamale and Dangme East District in Ghana showed higher prevalence of 63% and 66%, respectively [22, 52], which was higher than our study prevalence, indicating a concern of high anaemia and the need to intensify its reduction in pregnancy [52].

We observed better Hb outcomes with age, similar to studies in Bangladesh, China, and Ghana [57–59]. However, Kwabre East Municipality of Ghana reported a higher risk of anaemia in older women, and no variation of age and Hb was recorded by Akowuah et al. and Lumbanraja et al. [59, 60].

*P. falciparum* infection was associated with decreased Hb levels in pregnant women, and age was strongly associated with increased Hb levels for ANC and at the point of delivery. As previously reported, the destruction of *P. falciparum*-infected erythrocytes during schizont rupture is linked to decreased Hb levels during pregnancy [17].

Our study observed age to be another factor associated with malaria, and this could be as a result of parity-related immunity [31] and a gradual build-up of immunity to *P. falciparum* infection in older women which reduces their infection risk compared to younger women [17].

We observed a significant association of malaria and anaemia, suggesting that malaria contributes to anaemia during pregnancy [16]. Using the logistic regression model, pregnant women infected with *P. falciparum* have higher odds of being anaemic. Furthermore, when anaemia was stratified (mild, moderate, and severe), malaria remained strongly associated with higher risk of severe anaemia. The odds of anaemia in pregnant women as they get older was lowered in our study.

An older population of pregnant women in BH than in AH indicated a higher number of primigravid women in AH (55%) than BH (42%). Younger women in our study chose to attend ANC earlier at AH compared to BH. Malaria prevalence at AH and BH at delivery was significantly lower than ANC, although participants at ANC were not the same as delivery. This report testifies the benefits of routine ANC program which includes malaria diagnosis and IPTp in ensuring healthy pregnancies and reduction in malaria prevalence [30]. Adherence to IPTp protocol in the study participants as a preventive treatment for malaria during pregnancy was commendable, especially with more than 2 doses [30].

We observed that IPTp was associated with increased Hb outcomes [52], and more than 2 doses of IPTp received during pregnancy led to improved Hb levels at the point of delivery, agreeing with previous studies by Wilson et al. in Ghana [61]. However, reports from Sekondi Takoradi in Ghana showed that Hb levels were not different from IPTp users and nonusers [62]. They associated this difference to the gradual emergence of resistance in *P. falciparum* parasite to IPTp as has been observed by others [46, 62, 63]. Inadequate malaria immunity and compromised drug quality [52] were also identified as factors that could influence Hb. IPTp played a protective role in preventing anaemia during pregnancy. When adjusted for age, this protective role still remained significant. Our findings are similar to observation of better Hb outcomes and IPTp by Agyeman et al. [52].

Urogenital schistosomiasis is another parasitic infection known to be associated with anaemia in pregnancy [18]. Its prevalence of 4% at ANC and 2% at delivery was within the range (0.30-4.53%) observed by others in northern region and Dangme East District of Ghana [21, 22, 64]. Our study did not find an association between *S. haematobium* infections and anaemia, even though previous studies have reported an association [18, 22, 24]. Tay et al. found a significant association between *S. haematobium* infections and anaemia [22], but infection intensities were not reported in their study to suggest if intensities played a role in the anaemia observed [22]. We are of the view that no association between *S. haematobium* infection on Hb levels could result from lack of statistical power due to the low number of *S. haematobium* infections (*n* = 25) observed in our study at ANC. Low *S. haematobium* prevalence could be related to age of study participants.

Although schistosomiasis affects all age groups, there is a higher risk of infection in school-aged children, who harbor peak infections [35, 65–68] and are actively and constantly engaged in water contact activities such as swimming and fetching water for domestic purposes. Individuals aged below 16 years have been identified as the main group that actively shed *S. haematobium* eggs in most endemic communities [69, 70]. Typically, most of the participants were older than 20 years when prevalence of schistosomiasis is lowest compared to younger children (<20) who are more exposed and/or susceptible [71, 72]. This could explain the low intensities of infection observed in our study. Another reason for age variation with schistosomiasis could be related to immunity. As has been found previously, older women are capable of developing robust immunity which tends to lower their risk of reinfection [73]. It would not be surprising if these women may have been previously treated during MDA by Volta River Authority (VRA), Ministry of Health (MOH), and Ghana Health Service (GHS) before their current pregnancy, ensuring lowered *S. haematobium* prevalence [74]. The challenge for pregnant women is their exemption from MDA, although they are an important risk group who could be fueling schistosomiasis transmission cycle.

We observed that 96% of pregnant women had low intensity *S. haematobium* infections and therefore could not determine *S. haematobium* intensity and its association with anaemia. Nonetheless, the only high infection intensity (61 eggs/10 ml) recorded the lowest Hb level, agreeing with observations from other studies [24, 72, 75, 76]. Anaemia due to schistosomiasis results from blood loss as eggs with terminal spines pass through the urogenital tract in *S. haematobium* infection [24, 77, 78], leading to low Hb levels in the host.

We confirm in Ghana that while there is routine preventive treatment of malaria in pregnancy, routine diagnosis and anthelminthic treatment for pregnant women after their first trimester are yet to be implemented. Women aged 18-25 years living in endemic areas live almost a quarter of their reproductive lives being pregnant and more than 50% of reproductive lives lactating [6]. Suspension of anthelminthic treatment for a year or more could have severe consequences on morbidity and quality of life for these women [6, 25].

Starting a program at ANC to routinely diagnose and treat pregnant women could improve Hb levels as previous MDA among pregnant women reduced anaemia by 23% [18, 23]. Furthermore, deworming of pregnant women has been associated with a 14% reduction in neonatal death risk proportionally in low and high transmission countries [79].

## 5. Study Limitations

At delivery, we were faced with a difficulty of collecting urine samples from pregnant women, but an effort was made to recruit as many participants as possible. Urogenital schistosomiasis prevalence was estimated by microscopy which has a lower sensitivity compared to molecular methods; therefore, we anticipate that a number of schistosomiasis cases may have been missed in low intensity infections in our study population.

## 6. Conclusion

We report that anaemia is still a concern for pregnant women at ANC and delivery. *P. falciparum* infection was associated with low Hb levels with increased odds of becoming anaemic in pregnancy. However, more than two IPTp doses administered during pregnancy were associated with increased Hb levels among pregnant women. *S. haematobium*-related anaemia was not significant in our study. While routine diagnosis and treatment for PAM were implemented in our study hospitals, no evidence of routine diagnosis and treatment was seen for schistosomiasis during ANC. We suggest routine diagnostic tools which are rapid, sensitive, and cheaper as well as treatment of *S. haematobium* infections in endemic areas to reduce schistosomiasis during pregnancy.

## Figures and Tables

**Table 1:** Characteristics of participants during ANC and delivery.

Variable	Study site	*N*	Mean ± SD or *N* (%)	*p* value
ANC				
Age (years)	AH	393	25.96 ± 6.5	0.005^∗^
	BH	314	27.32 ± 6.1	
Primigravidae/multigravidae (%)	AH	393	124/269 (46.1)	0.227
	BH	314	90/224 (40.2)	
Gestational age (weeks)	AH	388	14.56 ± 7.1	0.002^∗^
	BH	307	16.19 ± 7.0	
Bed net use (*N*, %) (yes)	AH	393	359/393 (91.3)	0.398
	BH	313	289/313 (92.3)	
Hb	AH	393	10.618 ± 1.5	0.002^∗^
	BH	314	10.975 ± 1.4	
*S. haematobium* (*N*, %)	AH	348	14/348 (4)	0.689
	BH	314	11/324 (3.4)	
*P. falciparum* (*N*, %)	AH	357	101/357 (28.3)	0.008^∗^
	BH	320	59/320 (18.4)	

Delivery				
IPTp use (%)	AH	329	314/329 (95)	0.020^∗^
	BH	114	114//114 (100)	
Bed net usage (*N*, %) (yes)	AH	248	248/248 (100)	0.455
	BH	111	111/111 (100)	
Hb (g/dl)	AH	329	10.85 ± 1.1	0.913
	BH	114	10.87 ± 1.5	
*P. falciparum* (*N*, %)	AH	277	40/277 (14)	0.003^∗^
	BH	102	4/102 (4)	

^∗^Significance. *N* (%), total number of participants, and percentage are in parenthesis. Mean ± SD: mean ± standard deviation. Total prevalence of *S. haematobium* at delivery was 3/192 (1.6%). Primigravid, first pregnancy; multigravid, more than one pregnancy.

**Table 2:** Anaemia status of participants.

Anaemia status (Hb g/dl)	ANC			Delivery		
	Adidome (*N*, %)	Battor (*N*, %)	*p* value	Adidome (*N*, %)	Battor (*N*, %)	*p* value
Normal	175 (44.7)	167 (53.2)		166 (50.3)	60 (51.7)	
Mild	104 (26.5)	72 (22.9)	0.015^∗^	124 (37.6)	27 (23.3)	0.002^∗^
Moderate	105 (26.8)	75 (23.9)		39 (11.8)	29 (25.0)	
Severe	8 (2.0)	0		1 (0.3)	0	
Total	392	314		330	116	

^∗^Statistically significant. At ANC and delivery, at a threshold of 95% confidence, anaemia status varied between two hospitals: ANC (*p* = 0.015) and delivery (*p* = 0.002). Anaemia status of pregnant women was determined using WHO guidelines into normal (>11 g/dl), mild (10-10.99 g/dl), moderate (7.0-9.9 g/dl), and severe (<7 g/dl) groups.

**Table 3:** Factors influencing Hb level in pregnant women at ANC and delivery.

Variables	Linear regression models (Hb levels of g/dl)	SE	*p* value
	Coefficient (CI 95%)		
ANC			
*P. falciparum*	-0.48 (-0.78, -0.18)	0.15	0.002^∗^
*S. haematobium*	-0.46 (-1.18, 0.27)	0.37	0.215
Study area	0.10 (-0.16, 0.35)	0.13	0.461
Age	0.05 (0.03, 0.07)	0.01	<0.0001^∗^
Delivery			
IPTp (yes/no)	0.94 (0.31, 1.57)	0.32	0.003^∗^
Age	0.05 (0.03, 0.07)	0.01	0.004^∗^
IPTp dose [1]	0.67 (-0.12, 1.48)	0.41	0.097
IPTp dose (>2)	1.07 (0.36, 1.79)	0.36	0.003^∗^
Age	0.03 (-0.002, 0.065)	0.02	0.066

CI: confidence interval; SE: standard error. Asterisk (^∗^) indicates factors that significantly influence Hb levels at ANC or delivery at *p* < 0.05.

**Table 4:** Risk factors for anaemia during pregnancy (logistic regression).

Variable	ANC			Delivery		
	aOR	(95% CI)	*p* value	aOR	(95% CI)	*p* value
Malaria	1.92	(1.00, 3.67)	0.040^∗^	NS	NS	NS
Maternal age	0.95	(0.93, 0.98)	0.001^∗^	0.95	(0.89, 1.00)	0.04^∗^
IPTp	NA	NA	NA	0.73	(0.57, 0.93)	0.01^∗^

No significant difference between *S. haematobium* infection and anaemia. aOR: adjusted odds ratio; CI: confidence interval; NA: not available (no IPTp treatment during first ANC); NS: not statistically significant. ^∗^statistical significance.

## Data Availability

The data that support the findings of this study are available from the corresponding author (Naa Adjeley Frempong) upon reasonable request.
